# Supplementary material for: Generation of App knock-in mice reveals deletion mutations protective against Alzheimer’s disease-like pathology
Source: Nat Commun. 2018 May 4;9:1800. doi: 10.1038/s41467-018-04238-0 (PMC5935712; doi:10.1038/s41467-018-04238-0)
Supplement: Supplementary file 1 — Supplementary Information [file 41467_2018_4238_MOESM1_ESM.pdf]

Generation of App knock-in mice reveals deletion mutations protective against Alzheimer's disease-like pathology

Nagata et al.

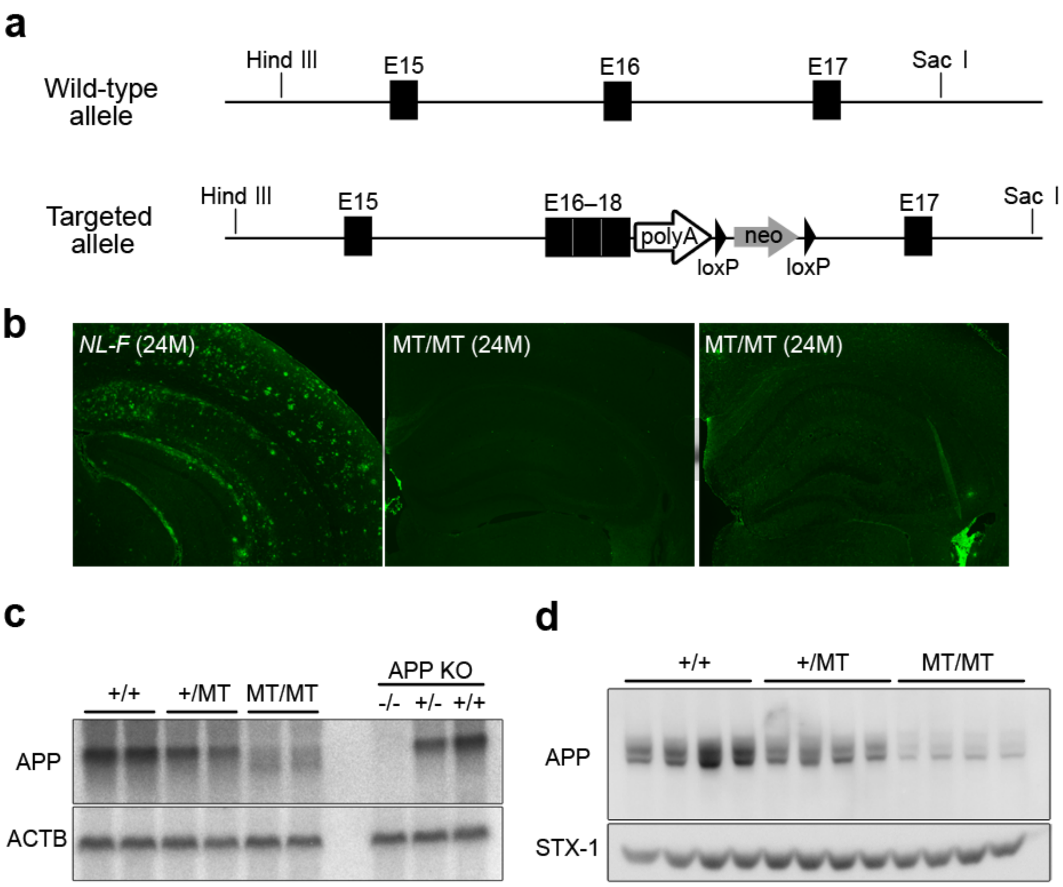

Supplementary Fig 1. Previous *App* knock-in model did not show Aβ accumulation in the brain.

(a) Wild-type allele of *App* gene and the targeted allele of *App* knock-in model carrying a humanized Aβ sequence as well as two clinically causative AD mutations in the Exons 16 or Exon 17. Note that The mutant lacks Introns 16 and 17 and most part of 3'-UTR. **(b)** In contrast to the $App^{NL\text{-}F}$ knock-in model[7], which carries the same pathogenic mutations and the introns and 3'-UTR, no Aβ deposition, was observed in the 24-month-old model without the introns and 3'-UTR (MT). **(c)** Northern blot analysis revealed that APP mRNA expression was clearly downregulated in the model without the introns and 3'-UTR (MT) in a gene dose-dependent manner. **(d)** APP expression was also decreased at protein levels. *ACTB* β-actin, *STX-1* Syntax-1.

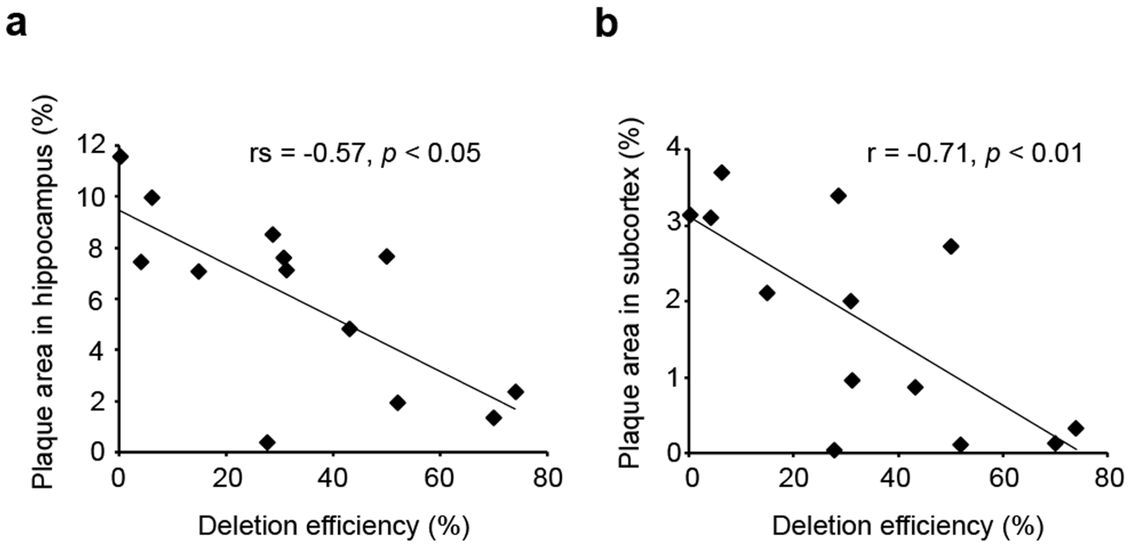

Supplementary Fig 2. Reduction of Aβ accumulation in *NL-G-F* ΔUTR brains

A negative correlation between Aβ accumulation and genomic deletion efficiency was observed in 6-month-old *NL-G-F* ΔUTR mouse (n = 13) hippocampus (a) and subcortex (b). *r* and *rs* indicate Pearson's correlation coefficient and Spearman's correlation coefficient, respectively.

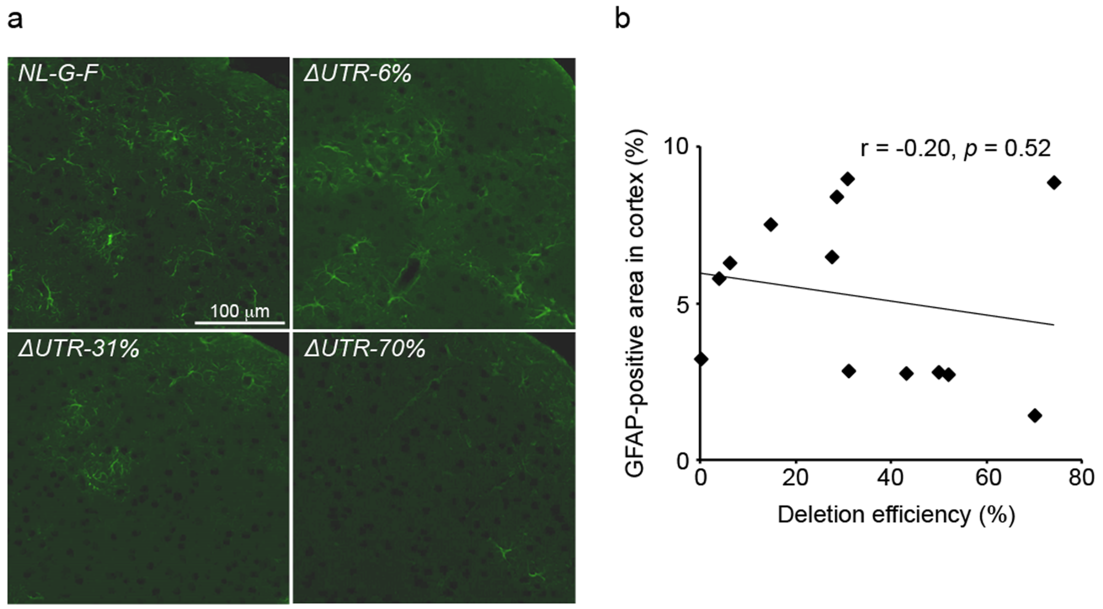

Supplementary Fig 3. Relationship between gliosis area and genome editing efficiency in the *NL-G-F* ΔUTR mouse cortex

(a) GFAP-positive astrocytes were visualized in 6-month-old unedited *NL-G-F* as well as *NL-G-F* ΔUTR mice. **(b)** A negative correlation between the GFAP-positive area and deletion efficiency was observed in 6-month-old *NL-G-F* ΔUTR mouse cortex (n = 13). r indicates Pearson correlation coefficient.

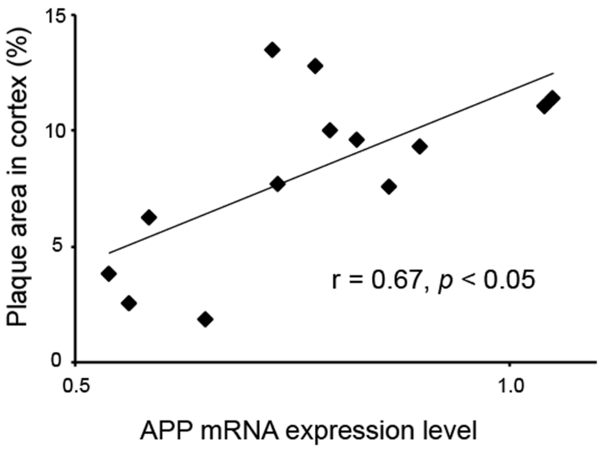

Supplementary Fig 4. Relationship between plaque area and APP mRNA expression level in the *NL-G-F* ΔUTR mouse cortex

Relative quantitative RT-PCR was performed in 6-month-old *NL-G-F* ΔUTR mouse cortex (n = 13). APP mRNA levels are normalized to GAPDH level. Values relative to control *NL-G-F* mice are shown as individual points. A positive correlation between the plaque area and APP mRNA level was observed in 6-month-old *NL-G-F* ΔUTR mouse cortexes. r indicates Pearson correlation coefficient.

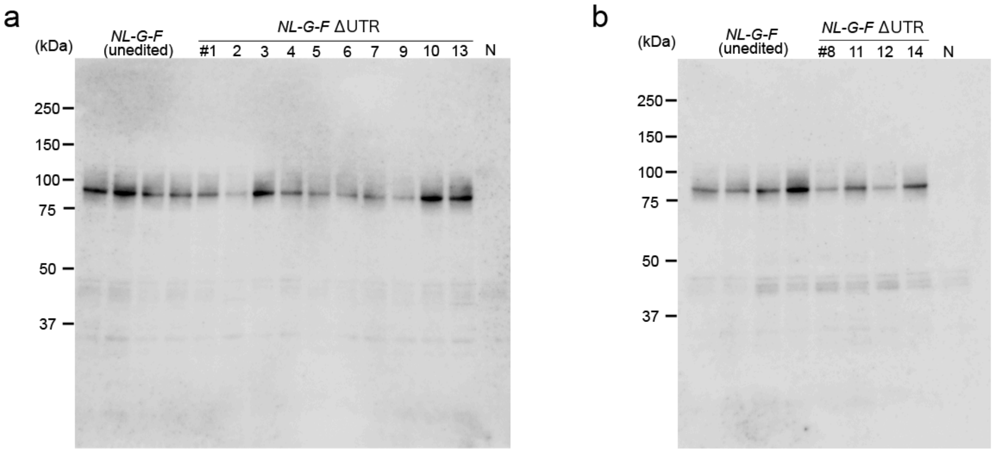

Supplementary Fig 5. Full-length images of western blots

Full-length images of APP western blots for 6-month-old male (a) and female (b) *NL-G-F* ΔUTR mouse brains. N represents APP KO mouse samples used as negative controls.

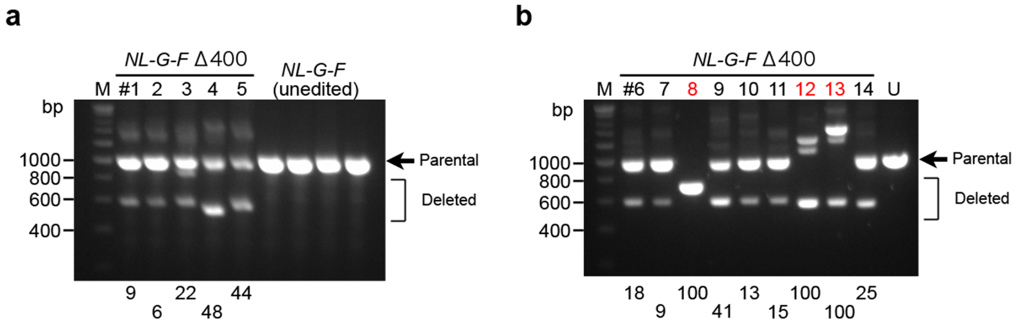

Supplementary Fig 6. PCR-based genotyping results of *NL-G-F* Δ400 mice.

Genotyping was performed using 6-month-old male (a) and female (b) *NL-G-F* Δ400 mouse brains. The deletion efficiency of each *NL-G-F* Δ400 mouse is represented on the bottom of each lane. Three mice (#8, 12, 13) were excluded from subsequent analyses because sequencing analyses showed relatively large insertions (#12, #13) or inverted sequence (#8) in their genome.

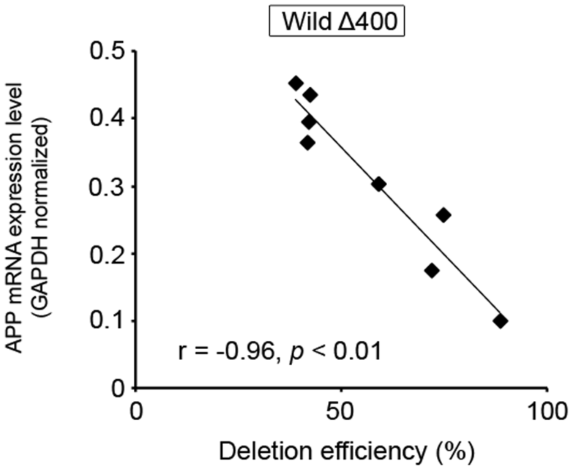

Supplementary Fig 7. Relationship between deletion efficiency and APP mRNA expression level in the wild-type ΔUTR mouse cortex

Negative correlation between APP mRNA expression and genome editing efficiency in wild-type Δ400 mouse brains (n = 8). r indicates Pearson correlation coefficient.

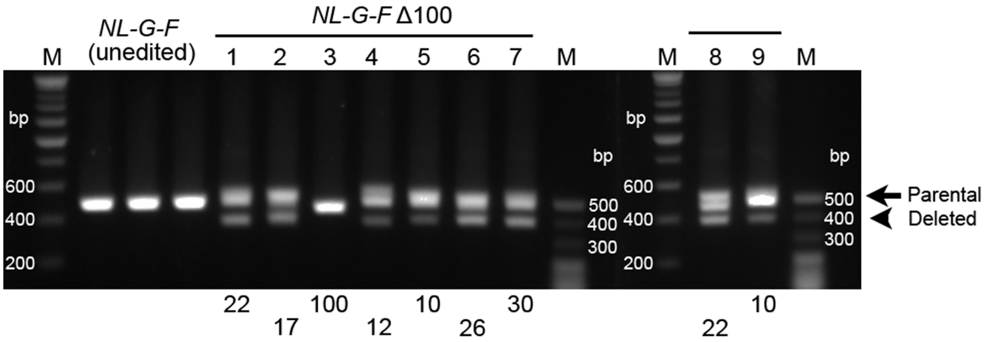

Supplementary Fig 8. PCR-based genotyping results of *NL-G-F* Δ100 mice.

Genotyping was performed using 6-month-old *NL-G-F* Δ100 mouse brains. The deletion efficiency of each *NL-G-F* Δ100 mouse is represented on the bottom of each lane. It was important to note that among the founder mice one mouse (#3) display homozygous deletion mutation (also see Fig. 3d).

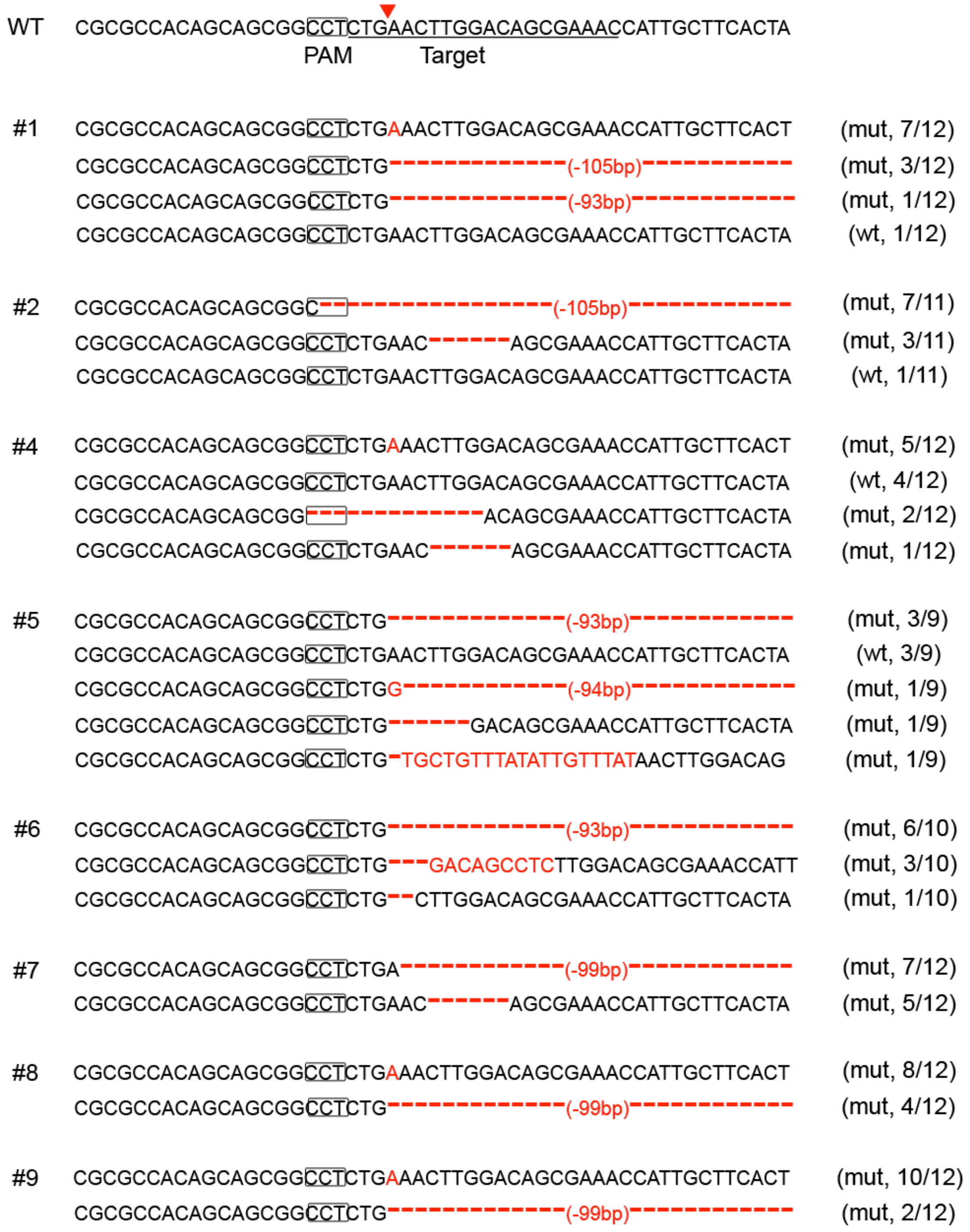

Supplementary Fig 9. Sequences of modified 52-bp elements in 8 founders

Colony sequencing was performed to determine the detailed genotypes of CRISPR/Cas9-injected founder mice (#1, 2, 4-9 from Fig. 3c, d). Deleted sequences and inserted sequences are shown in red dashes and red characters, respectively. The fractions on the right indicate the mutant reads number out of total reads number.

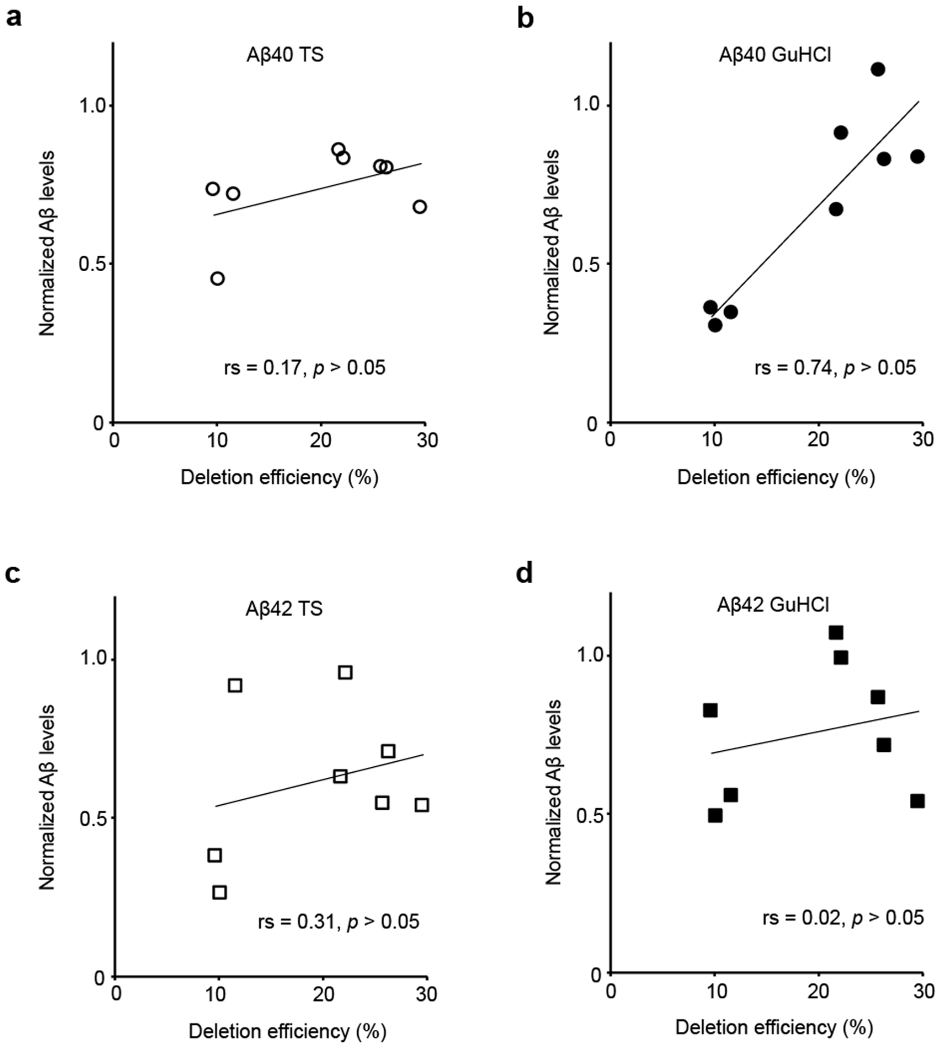

Supplementary Fig 10. Relationship between deletion efficiency and Aβ levels in *NL-G-F* Δ100 mice. $A\beta_{40}$ (a, b) and $A\beta_{42}$ (c, d) levels in both the Tris-HCl-buffered saline (TS) and GuHCl fractions were quantified by ELISA. No clear correlation between normalized Aβ levels and deletion efficiency in 6-month-old *NL-G-F* Δ100 mouse brains (n = 8). Note that one *NL-G-F* Δ100 mouse (#3) were excluded in this correlation analysis due to its complete different genotype (i.e. homozygous 34-bp deletion).

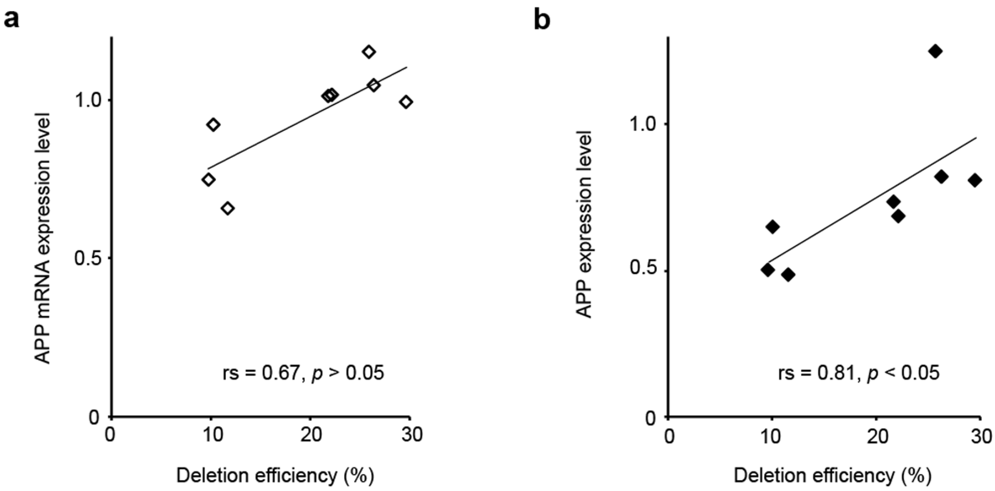

Supplementary Fig 11. Relationship between deletion efficiency and APP expression levels in *NL-G-F* Δ100 mice.

APP expression levels in *NL-G-F* Δ100 mice were assessed by quantitative PCR (a) or western blotting analysis (b). Note that one *NL-G-F* Δ100 mouse (#3) were excluded in this correlation analysis.

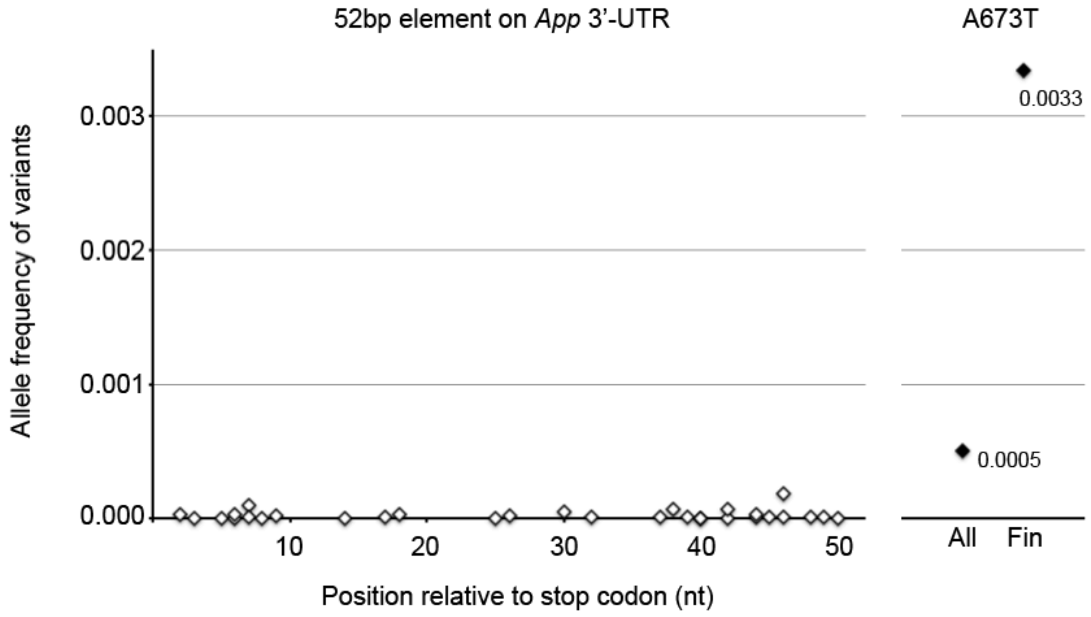

Supplementary Fig 12. Allele frequency of variants in *App* 3'-UTR and A673T missense mutation

Allele frequencies of variants were determined using publicly available variant databases (ExAC, http://exac.broadinstitute.org; gnomAD, http://gnomad.broadinstitute.org). The frequency of all variants in the 52-bp regulatory element on *App* 3'-UTR are quite low. In contrast, the allele frequency of a previously reported protective missense mutation (A673T) is relatively high, especially in the Finnish population (Fin).

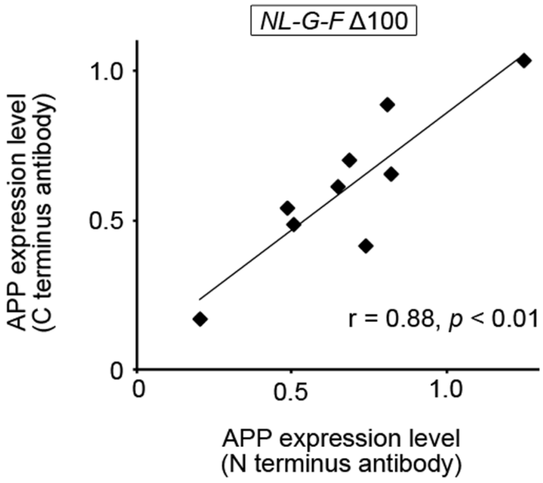

Supplementary Fig 13. Validation of western blotting results with two distinct APP antibodies. Western blotting analyses were performed using two distinct APP antibodies: N-terminal APP antibody and C-terminal APP antibody. *NL-G-F* Δ100 mouse brains were used as samples. The APP expression levels correlated well between the antibodies.

Name	Sequence (5'-3')	Usage
APP 3UTR Fw	tgcagcagaacggatatgag	genotyping
APP 3UTR Rev	aaagaaacgtcttgcctgga	
APP 3UTR short deleted Fw	ctctccaagatgcagcagaa	
APP 3UTR short deleted Rev	ccccatcgatttttaaagca	
APP 3UTR T7-sgRNA 1 Fw	ttaatacgactcactataggGTTTCGCTGTCCAAGTTCAG	sgRNA synthesis
APP 3UTR T7-sgRNA 2 Fw	ttaatacgactcactataggTCATAAGCACTTTTACGGGT	
APP 3UTR T7-sgRNA 3 Fw	ttaatacgactcactataggTATGCTTTAAAAATCGATGG	
APP 3UTR T7-sgRNA 4 Fw	ttaatacgactcactataggGGGATGCTTCTTGTGAACGT	
APP 3UTR T7-sgRNA 5 Fw	ttaatacgactcactataggTCCGTTTATTTACTCACCCT	
APP 3UTR T7-sgRNA 6 Fw	ttaatacgactcactataggAGCACAGCTGTCAAAAGCCG	
T7-sgRNA Rev	aaaagcaccgactcggtgcc	
sgRNA-1_OT1 Fw	aaacgtccatttcagcatca	off-target analysis
sgRNA-1_OT1 Rev	tcccttttggaaaagaggttt	
sgRNA-1_OT2 Fw	caggatcttggcctttagcc	
sgRNA-1_OT2 Rev	ctggctgctgatttgtgtgt	
sgRNA-1_OT3 Fw	gctccttcttccctgtagcc	
sgRNA-1_OT3 Rev	tctctgctgcttacgctcaa	
sgRNA-1_OT4 Fw	tacaggccccacaagcatta	
sgRNA-1_OT4 Rev	ctgggttcctacatggcatt	
sgRNA-1_OT5 Fw	gagattgcctgccaatgttt	
sgRNA-1_OT5 Rev	gctcgggatacaagagttgc	
sgRNA-2_OT1 Fw	gctcactgaaaacccactcc	
sgRNA-2_OT1 Rev	tcagaggatggttcaccaca	
sgRNA-2_OT2 Fw	ctgctgagggcatcctgta	
sgRNA-2_OT2 Rev	tgcccagcctgttctaattt	

Supplementary Table 1. Primer sequences

Zygote Strain	Cas9/sgRNA-1/sgRNA-(2-6) (ng/µl)	sgRNA combinations	Injected Zygotes	Transferred Zygotes	Live-Born Pups	3'-UTR Deleted Founders/Pups (%)	Founder Name
NL-G-F	10/10/10	sgRNA-1, 2	103	75	24	4 (16.7)	*NL-G-F* ΔUTR
NL-G-F	30/30/30	1, 2	104	80	25	10 (40.0)	
NL-G-F	0/0/0		105	86	35		*NL-G-F* (unedited)
NL-G-F	10/10/30	1, 3	130	86	33	9 (27.3)	*NL-G-F* Δ400
NL-G-F	10/10/30	1, 4	130	103	29	5 (17.2)	
C57BL/6	10/10/30	1, 3	75	46	20	3 (15.0)	WT Δ400
C57BL/6	10/10/30	1, 4	74	47	17	5 (29.4)	
C57BL/6	10/0/0		66	46	19		WT (unedited)
NL-G-F	10/10/30	1, 5	130	82	37	7 (18.9)	*NL-G-F* Δ100
NL-G-F	10/10/30	1, 6	129	79	16	4 (25.0)	
NL-G-F	10/0/0		50	32	15		*NL-G-F* (unedited)

Supplementary Table 2. Efficiency of CRISPR/Cas9-mediated *App* 3'-UTR deletion

sgRNA-1	Target Sequence	PAM (NGG)	Mismatches	Locus	Mutation/Total Number
ON	GTTTCGCTGTCCAAGTTCAG	AGG		Chr16:84955284-84955306	
OT-1	GTTGAGCTGTCCAAATTCAG	GGG	3	ChrX:120201476-120201498	0/14
OT-2	GTTTTGTTGTCCAAGTTCAG	GAG	3	Chr1:53630507-53630529	0/14
OT-3	GTTTCACTGTCCAGGTTCTG	TGG	3	Chr7:112574971-112574993	0/14
OT-4	GTTTCCCTGTCAAAGTCCAG	GGG	3	Chr13:74061778-74061800	0/14
OT-5	GTTTCTCTGTCCAACATCAG	TGG	3	Chr6:124413763-124413785	0/14
**sgRNA-2**					
ON	TCATAAGCACTTTTACGGGT	GGG		Chr16:84954586-84954608	
OT-1	TCAAAAGCTCTTTTACGGGC	TGG	3	Chr5:42759728-42759750	0/14
OT-2	TCATAAGTACTGTTACAGGT	TGG	3	Chr7:52434783-52434805	0/14

Supplementary Table 3. Off-target analysis in *App* 3'-UTR deleted founders
